# Basic mechanisms of rTMS: Implications in Parkinson's disease

**DOI:** 10.1186/1755-7682-1-2

**Published:** 2008-04-15

**Authors:** Oscar Arias-Carrión

**Affiliations:** 1 Experimental Neurology, Philipps University, D-35033 Marburg, Germany

## Abstract

**Background:**

Basic and clinical research suggests a potential role for repetitive transcranial magnetic stimulation (rTMS) in the treatment of Parkinson's disease. However, compared to the growing number of clinical studies on its putative therapeutic properties, the studies on the basic mechanisms of rTMS are surprisingly scarce.

**Results:**

Animal studies have broadened our understanding of how rTMS affects brain circuits and the causal chain in brain-behavior relationships. The observed changes are thought to be to neurotransmitter release, transsynaptic efficiency, signaling pathways and gene transcription. Furthermore, recent studies suggest that rTMS induces neurogenesis, neuronal viability and secretion of neuroprotective molecules.

**Conclusion:**

The mechanisms underlying the disease-modifying effects of these and related rTMS in animals are the principle subject of the current review. The possible applications for treatment of Parkinson's disease are discussed.

## Background

The clinical motor dysfunction in Parkinson's disease (PD) is primarily the consequence of a progressive degeneration of dopaminergic neurons in the substantia nigra of the nigrostriatal pathway. The degeneration of this tract provokes a depletion of dopamine in the striatum, where it is required for normal motor function [1]. Restoring dopamine levels with L-dopa is still the most widely used treatment of PD. However, despite conflicting data, L-dopa is suspected to exert neurotoxic properties that can accelerate the loss of dopaminergic neurons [2]. In addition, it elicits marked dyskinesias, and its therapeutic efficacy gradually wanes over years of exposure [2]. Although the dopamine precursor is effective in the short-term in relieving motor dysfunction, it does not stop the progressive disappearance of dopaminergic neurons, encouraging interest in alternative therapeutic strategies [2,3].

Recent studies suggest a potential role of repetitive transcranial magnetic stimulation (rTMS) in different neuropsychiatric diseases and have been used to investigate almost all areas of cognitive neuroscience [for review see refs [4,5]. The use of rTMS in clinical neurophysiological studies is highly advanced and has been reviewed elsewhere [which can be found in refs [5-8]]. rTMS is a non-invasive brain stimulation technique that can produce lasting changes in excitability and activity in cortical regions underneath the stimulation coil (local effect), but also within functionally connected cortical or subcortical regions (remote effects) [4-8]. Since the clinical presentation of PD is related to abnormal neuronal activity within the basal ganglia and cortical regions, including the primary motor cortex, the premotor cortex and the prefrontal cortex [1], several studies have used rTMS to improve brain function in PD [6,7]. The basic mechanisms of rTMS in the context of pathophysiology of PD are the principle subject of the current review.

### rTMS as a technique for noninvasive stimulation of the human brain

Electromagnetic induction was described by Michael Faraday. This principle, formulated mathematically by James C. Maxwell, states that fluctuating magnetic fields can induce electric current in conductors placed nearby. Recently, this principle has been applied to induce electric current in the adult human brain, in a technique known as transcranial magnetic stimulation (TMS) [for review see refs [4,5]]. A variant of TMS, in which the stimulus can be repeated for a few seconds at frequencies up to 50 Hz, is referred to as rTMS [4-6]. rTMS is an experimental tool for stimulating neurons via brief magnetic pulses delivered by a coil placed on the scalp. rTMS can non-invasively interfere with neural functions related to a target cortical area with high temporal accuracy [4-6]. The key features of the technique are that rTMS machine delivers a large current in a short period of time – the current in the rTMS coil then produces a magnetic field which, if changing rapidly enough, will induce an electric field sufficient to stimulate neurons or change the resting membrane potential in the underlying cortex [4-8]. In short, rTMS can be used to induce a transient interruption of normal brain activity in a relatively restricted area of the brain [4-8]. Because of these unique and powerful features, rTMS has been popular in various fields, including cognitive neuroscience and clinical application [for review see refs [4-7]]. However, despite its utility, the mechanisms of how rTMS stimulates neurons and interferes with neural functions are still unknown.

### Cellular and molecular mechanisms of rTMS

**Figure 1:**
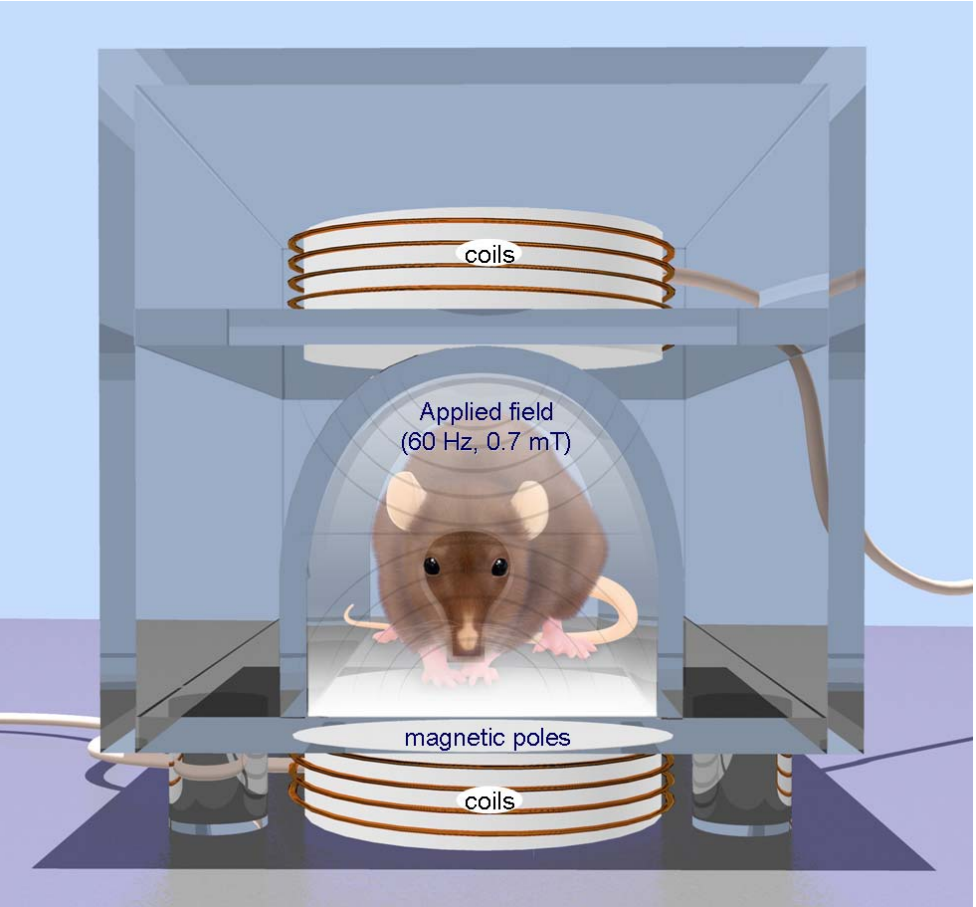
**Repetitive transcranial magnetic stimulation (rTMS) is a technique for noninvasive stimulation of the adult brain.** Stimulation is produced by generating a brief, high-intensity magnetic field by passing a brief electric current through a magnetic coil. Compared with the growing number of clinical trial with rTMS, there are surprisingly few animal studies on its basic mechanisms of action, constraining the ability to perform hypothesis-driven clinical studies.

Animal studies have broadened our understanding of how rTMS affects brain functioning (Fig. 1). There is evidence that rTMS causes changes in neuronal circuits as reflected by behavioral changes [4-8]. These alterations include regional changes in the release of neurotransmitters, transsynaptic efficiency, signaling pathways and in gene transcription.

A possible mechanism by which rTMS exerts and effect is through gene induction [8-10]. Different studies support that rTMS modulates the expression of immediate early genes such as c-fos and *c-jun *[8-10]. These genes are activated transiently and rapidly in response to a wide variety of cellular stimuli. They represent a standing response mechanism that is activated at the transcription level in the first round of response to stimuli, before any new proteins are synthesized [8-10]. A single rTMS train increased c-fos mRNA in the paraventricular nucleus of the thalamus, and to a lesser extent in the frontal and cingulate cortices, but not in the parietal cortex [9]. In contrast to these acute effects, 14 daily rTMS treatments increased c-fos mRNA in the parietal cortex [10]. No change was seen in Brain-derived neurotrophic factor (BDNF) mRNA expression [10]. Another study found that a longer treatment (5-day series separated by 2-day intervals for 11 weeks) significantly enhanced BDNF mRNA in the hippocampus and the parietal and piriform cortices [11]. The BDNF is the most abundant and widely distributed neurotrophin in the CNS and plays regulatory roles in many neuronal functions including survival, neurogenesis, and synaptic plasticity. Effects on neurotrophic factors could possibly explain preliminary findings of neuroprotective and neuroplastic effects of rTMS, such as mossy fiber sprouting in the hippocampus following chronic rTMS [12]. Possible effects of rTMS on neurotrophic factors might be relevant to new theories about the mechanisms of action of antidepressant medications [13].

Importantly, specific effects of rTMS on neurotransmitter system have been repeatedly reported. Acute treatment with rTMS in rodents modulates monoamine content and turnover [14,15], but no effects on neurotransmitter levels or metabolites have been reported after chronic stimulation [16]. Shortly after rTMS, dopamine is reported to be reduced in the frontal cortex and increased in the striatum [14] and the hippocampus [14,15]. Increased serotonin (5-HT) in the hippocampus was found in brain homogenates with HPLC [14], but not in an *in vivo *microdialysis study [15]. Reductions in arginine vasopressin release and increases in taurine, aspartate, and serine were reported in the hypothalamic paraventricular nucleus with rTMS [15].

The dopaminergic system might therefore be one of the primary candidate neurotransmitter systems which are directly and selectively modulated by rTMS of frontal brain regions. It ventral tegmental area (VTA) and the substantia nigra, i.e. the regions of origin of the mesolimbic and mesostriatal dopaminergic pathways [17]. These neuroanatomical connections may explain how stimulation of frontal brain regions enhances dopamine efflux in axon terminal areas originating from mesencephalic dopaminergic cell groups. Apart from the hippocampus, the ventral (i.e. nucleus accumbens) and dorsal striatum receive dense dopaminergic projections from the VTA and substantia nigra respectively, [18,19] and therefore might be candidate regions for possible rTMS-induced changes in interneuronal communication. Consistent with the hypothesis that stimulation of frontal brain regions by rTMS may increase dopaminergic neurotransmission in areas other than the hippocampus, it has been reported that direct electrical stimulation of the prefrontal cortex enhances dopamine release in the dorsal striatum and nucleus accumbens [20,21].

The neurochemical changes induced by the exposure to rTMS *in vivo *are preserved and expressed in the tissue *in vitro*. rTMS initiates the biosynthesis of new molecules which persist in the tissue beyond the period of stimulation [22,23]. The activation of a rat's brain with rTMS enhanced the magnitude of long-term potentiation (LTP) recorded *in vitro *[24] and made the hippocampal slices prepared from exposed brains much more resistant to ischemic damage [25,26]. This data suggest a protective effect of BDNF for synaptic transmission after transient forebrain ischemia.

Given the beneficial effects of rTMS in the treatment of affective disorders, the mesolimbic dopaminergic system is of particular interest as it comprises the major components of the neural circuitry of reward and incentive motivation [27]. With respect to PD, activation of the mesostriatal dopaminergic pathway is likely to be a possible candidate mechanism behind the therapeutic effects of rTMS that have been reported [28-30].

Several studies in humans have shown that acute and chronic rTMS treatments of the frontal cortex improve the symptoms of PD, which is thought to result from subcortical dopamine dysfunction [31]. Interestingly, acute rTMS treatment of the prefrontal cortex (PFC) in humans was demonstrated by functional neuroimaging to induce a release of endogenous dopamine in the ipsilateral dorsal striatum [32]. Animal studies have shown that descending pathways from the frontal cortex modulate dopamine release in subcortical areas, such as the striatum [33]. Modulation of dopamine release may be relevant to the pathophysiology of PD [31].

### Neurogenesis in the adult brain

**Figure 2:**
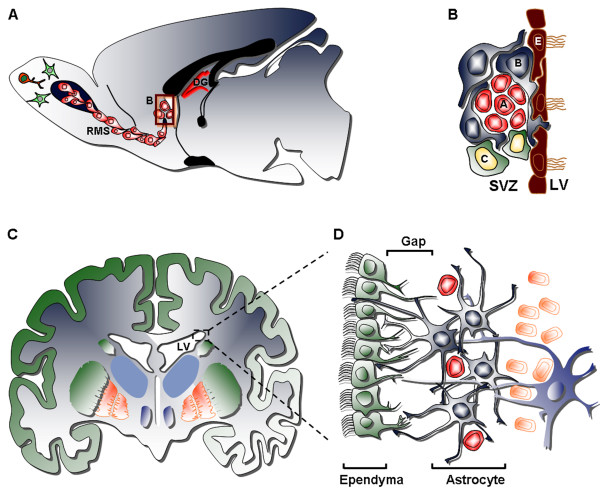
**The anatomy of the neurogenic subventricular zone in the adult rodent and human brain.** (A) Sagittal view of a rodent brain showing the sites of neurogenesis in the subventricular zone/olfactory bulb (SVZ/OB) system. (B) Schematic drawing of the composition and cytoarchitecture of the adult rodent SVZ. (C) Coronal view of the adult human brain showing the basal ganglia and lateral ventricles. (D) Schematic drawing depicting the cellular composition and cytoarchitecture of the adult human SVZ, consisting of four layers: Layer I – ependymal cell layer (green), Layer II – hypocellular gap, Layer III – astrocytic ribbon, containing astrocytes and migrating neuroblasts, Layer IV – transitional zone, containing oligodendrocytes and separating the SVZ from the striatum rich in neurons. RMS: rostral migratory stream; DG: dentate gyrus; LV: lateral ventricle.

The dogma that the adult mammalian brain is incapable of cellular self-repair has finally been overcome, when the generation of new neurons in the adult brain was described for the subventricular zone (SVZ) as well as for the dentate gyrus subgranular zone (SGZ) of the hippocampus [3,34-36] (Fig. 2A). The SVZ is an important germinal layer that forms during development adjacent to the telencephalic ventricular zone. This layer is most prominent in the lateral wall of the lateral ventricle facing the developing ganglionic eminences. The SVZ contains the largest pool of dividing neural progenitors/stem cells (NSCs) in the adult brain of all mammals, including humans [3,34-36]. In rodents, the adult SVZ contains four cell types defined by their morphology, ultrastructure, molecular markers and electrophysiological properties: 1) migrating neuronal progenitor cells (type A cells), 2) SVZ protoplasmic astrocytes (type B cells), 3) transit amplifying progenitor cells (type C cells), and 4) ependymal cells (type E cells) that separated the SVZ from the ventricular cavity, whose function is to circulate the cerebrospinal fluid (Fig. 2B) [3,34-36]. The organization of the adult human SVZ is significantly different compared to that of rodents (Fig. 2C). In adult rodents, SVZ astrocytes (Type B cells) are located next to the ependymal layer. In contrast, in the adult human brain, SVZ astrocytes are not found adjacent to the ependymal cells (Fig. 2D). Instead, the cell bodies of human SVZ astrocytes accumulate in a band or ribbon separated from the ependymal layer by a gap that is largely devoid of cells [37].

Different growth/trophic factor and cytokines have been identified *in vivo *as modulators of the numeric expansion and as fate determinants of NSCs, such as epidermal growth factor (EGF), transforming growth factor α (TGF-α), fibroblast growth factor (FGF-2), nerve growth factor (NGF), BDNF, glial cell line-derived neurotrophic factor (GDNF), basic fibroblast growth factor (bFGF), insulin-like growth factor-1 (IGF-1), erythropoietin (EPO) and others [3,34-36]. These agents, however, appear to act solely as mitogens without changing the fate determination of the NSCs and their progeny.

Studies in various animal disease models have convincingly shown that the SVZ can respond to insults in the adult brain by producing new progenitor cells that can migrate to sites that have been affected by neurodegenerative pathology or brain injury [3,36]. In response to neurodegeneration in Huntington's disease, epilepsy, multiple sclerosis and stroke, there is an upregulation of progenitor cell production, cytokine levels and migratory proteins in the SVZ, leading to an increase in the number of adult-born neurons. By contrast, in Alzheimer's disease and PD there are fewer proliferating cells in the SVZ [3,36].

### Neurogenesis and rTMS

With regard to the development of a cell replacement therapy in PD, it is intriguing to know that dopaminergic neurons are constitutively regenerated by adult neurogenesis in the olfactory bulb of the adult brain [3,34-37]. These neurons are born in the SVZ and migrate via the rostral migratory stream to the olfactory bulb, where they integrate as interneurons in the glomerular layer [3,34-37]. A detailed understanding of the molecular signals governing the proliferation, targeted migration and dopaminergic differentiation of these precursor cells may offer the exciting perspective that the endogenous NSCs in the SVZ may ultimately be instrumentalized for non-invasive replacement of the degenerating nigrostriatal system by autologous dopaminergic neurons [3].

**Figure 3:**
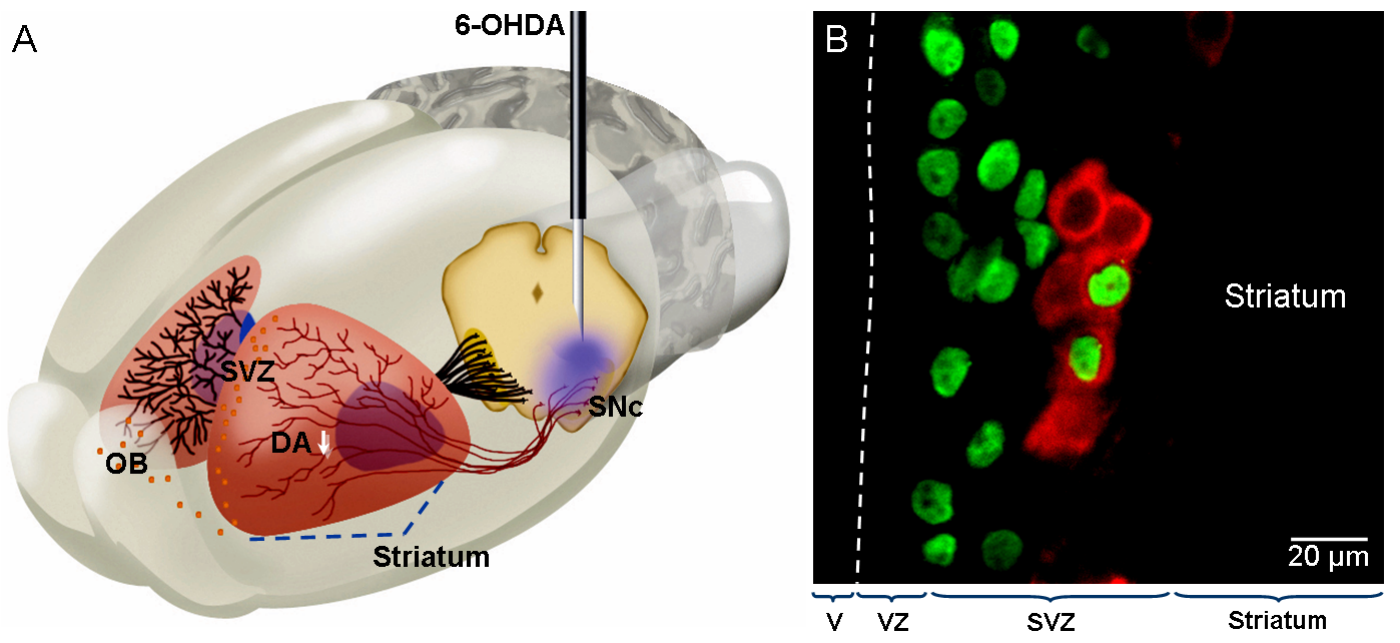
**Neurogenesis and rTMS.** (A) Dopaminergic neurons reside in the substantia nigra pars compacta (SNc), which is located in the ventral midbrain, and send axonal projections to the striatum, which is situated in the forebrain. Neural stem cells are located in the adult SVZ, immediately adjacent to the striatum, with is rich in dopaminergic afferents from the SNc. In the 6-OHDA animal model of Parkinson's disease, the nigral dopaminergic neurons are destroyed unilaterally by means of a stereotactic injection of the toxin, as indicated by the needle. The consecutive depletion of dopamine (DA↓) in the striatum leads to a decreased proliferation of progenitor cells in the SVZ. (B) In rats with unilateral 6-OHDA lesion of the SNc, rTMS induces an *in situ *differentiation of SVZ-derived precursors in dopamine-producing neurons. Some nucleus marked with BrdU (green) colocalize with the cytoplasmatic TH (red). OB: olfactory bulb; V: ventricle; VZ: ventricular zone.

Dopaminergic innervation and signalling in the SVZ profoundly increases the SVZ's proliferative capacity. A recent study used a 6-hydroxidopamine (6-OHDA) lesion model of PD, in which the medial forebrain bundle (also know as the nigrostriatal pathway) is destroyed by an excitotoxin, and measured proliferation in the SVZ [38] (Fig. 3A). The number of proliferating cells was found to have decreased by approximately 40%, as measured by bromodeoxyuridine (BrdU)-positive cells counts. Furthermore, this study demonstrated that the amount of proliferation in the SVZ after a 6-OHDA lesion was proportional to the amount or remaining dopaminergic innervation in the striatum [38]. After the dopamine precursor L-DOPA was administered, normal proliferation levels were restored in the side ipsilateral to the lesion, however, on the contralateral side, L-DOPA had no effect [38]. In a hypothesis-based attempt to identify such factors, intrastriatal infusion of TGFα in a rodent model of PD has been shown to induce massive proliferation and migration of cells from the SVZ toward the TGFα infusion site [39]. This treatment has been reported to result in increased numbers of dopaminergic-like neurons in the striatum. In behavioral experiments, there was a significant reduction of apomorphine-induced rotations in animals receiving the TGFα. However, a more recent attempt to replicate this finding has reproduced the precursor cell immigration, but failed to show a neuronal phenotype of the newborn striatal cells [40]. In this later study, intrastriatal TGFα infusion induced a significant increase in SVZ proliferation and substantial migratory waves of nestin-positive progenitor cells from the adult SVZ into the striatum of dopamine-depleted rats, but there was no dopaminergic differentiation of the newborn neurons [40]. In a similar way, infusion of BDNF into the lateral ventricle of the adult rat has been reported to induce migration of SVZ-derived neuroblasts into the parenchyma of the striatum [41].

In sharp contrast, there appears to be a reduction in precursor cell proliferation in the SVZ of PD patients and experimental animals as a consequence of dopamine depletion [3,38]. Recent studies have suggested that 60 daily rTMS treatments (60 Hz, 0.7 mT; Fig. 1) induce an *in situ *differentiation of SVZ-derived precursors in dopamine-producing neurons in rats with unilateral 6-OHDA lesions of the substantia nigra (Fig. 3) [42,43]. In behavioral experiments, there was a significant reduction of amphetamine-induced rotations in animals receiving the rTMS. The number of new dopaminergic cells was correlated with better locomotor activity (Fig. 3B). Patch-clamp studies suggested that a small percentage of SVZ-derived dopaminergic-like cells exhibited the electrophysiological properties of mature dopaminergic neurons and presented spontaneous postsynaptic potentials [43]. Further work is required to identify the key molecular players regulating this phenomenon, in order to develop novel cell therapies for PD based on rTMS.

On the other hand, it has been demonstrated that cerebral ischemia increases neurogenesis both in the SGZ and in the SVZ of the adult brain [44-46]. This increase has been associated with the activation of the NMDA receptor [47]. The neuronal precursors of the SVZ migrate to the ischemic zone of the adjacent striatum [45,46] and through the rostral migratory stream (RMS) and the lateral cortical stream to the ischemic zone of the cerebral cortex where the damaged neurons are differentiated and replaced [44-46]. Clinical trials using low-frequency rTMS applied to the unaffected hemisphere demonstrated decreased interhemispheric inhibition of the affected hemisphere with the associated behavioral changes in stroke patients [48,49]. Despite there being clinical evidence suggesting that electrical cortical DC stimulation to the affected hemisphere1 and low frequency rTMS to the unaffected hemisphere can modulate the cortical excitability and produce measurable hand motor improvement in stroke patients, the efficacy of high-frequency rTMS on the corticomotor excitability and the acquisition of motor skills in chronic stroke patients is being explored [48-50]. Although the effect of rTMS on neurogenesis in post-stroke  is not known, it is a study that should be explored.

## Conclusion

To date, experimental evidences suggest that rTMS induces changes in neurotransmitter release, transsynaptic efficiency, signaling pathways and gene transcription. Furthermore, recent studies suggest that rTMS induces neurogenesis, neuronal viability and secretion of neuroprotective molecules in an animal model of PD. rTMS represents a new strategy to regenerate the CNS enhancing neurogenesis in adults. A detailed understanding of the factors governing adult neurogenesis in vivo may ultimately lead to elegant cell therapies for PD and other neurodegenerative disorders by mobilizing endogenous NSCs to replace degenerated neurons. Finally, we must remember that however exciting the neurobiological mechanisms might be, the clinical usefulness of rTMS will be determined by their ability to provide patients with neurological disorders with safe, long-lasting and substantial improvements in quality of life.

## List of Abbreviations

rTMS, repetitive transcranial magnetic stimulation; PD, Parkinson's disease; CNS, central nervous system; TMS, transcranial magnetic stimulation; BDNF, brain-derived neurotrophic factor; S-HT, serotonin; HPLC, High-performance liquid chromatography; VTA, ventral tegmental area; LTP, long-term potentiation; PFC, prefrontal cortex; SVZ, subventricular zone; SGZ, subgranular zone; NSCs, neural progenitors/stem cells; EGF, epidermal growth factor; TGF-α, transforming growth factor α; FGF-2, fibroblast growth factor; NGF, nerve growth factor; GDNF, glial cell line-derived neurotrophic factor; bFGF, basic fibroblast growth factor; IGF-1, insulin-like growth factor-1; EPO, erythropoietin; 6-OHDA, 6-hydroxidopamine; BrdU, bromodeoxyuridine; L-DOPA, L-Dihydroxyphenylalanine; NMDA, N-methyl-D-aspartic acid; V, ventricle; VZ, ventricular zone.

## Competing interests

The author(s) declare that they have no competing interests.
